# Microbial spectrum and resistance of odontogenic abscesses - microbiological analysis using next generation sequencing

**DOI:** 10.1007/s00784-024-06097-0

**Published:** 2024-12-10

**Authors:** Felix Thol, Felix Benjamin Warwas, Nikolai Spuck, Franz-Josef Kramer, Nils Heim

**Affiliations:** 1 Department of Oral and Maxillofacial Plastic Surgery, University Medical Center of the University Bonn, Venusberg Campus 1, Building 11, 2. OG, D-53127 Bonn, Germany; 2 Institute of Medical Biometry, Informatics and Epidemiology, Medical Faculty, University of Bonn, Venusberg Campus 1, Building 11, D-53127 Bonn, Germany

**Keywords:** Odontogenic abscesses, Microbiome, Next-generation sequencing, NGS, Antibiotic resistance, Fungi

## Abstract

**Objectives:**

This study aimed to map the microbiome of odontogenic abscesses using next-generation sequencing (NGS) to identify bacterial and fungal species, as well as antibiotic resistances.

**Materials and methods:**

Wound swabs were taken from patients treated for odontogenic abscesses at the Department of Oral and Maxillofacial Plastic Surgery, University Hospital Bonn. NGS was used to analyze the swabs, and bioinformatic analysis assigned the genetic material to microorganism profiles and identified antibiotic resistances.

**Results:**

Bacteria were detected in all samples from 51 patients. Anaerobes were found in 50 swabs, while aerobic bacteria were detected in 30. Four of the five most common bacterial genera were anaerobes (Fusobacterium, Prevotella, Parvimonas, Porphyromonas). A median of eight genera were identified per swab. Infections were mostly polymicrobial; only one case was a monoinfection with Streptococcus. Aerobic bacteria were less frequent in patients who had prior antibiotic therapy (*p* = 0.015). Fungi were present in 52.9% of cases, with Malassezia (33.3%), Aspergillus (9.8%), and Candida (3.9%) being the most common. Antibiotic resistance was detected in 66.7% of patients, mainly against lincosamides, macrolides and tetracyclines. Fusobacterium showed a 25.9% resistance rate to clindamycin.

**Conclusions:**

The microbiome of odontogenic abscesses is polymicrobial, dominated by anaerobic bacteria, and more extensive than indicated by traditional cultural diagnostics. NGS provides detailed pathogen diagnostics, aiding in precise and individualized antibiotic therapy.

**Clinical relevance:**

Improved understanding of the bacterial and fungal spectrum, along with current resistance patterns of odontogenic abscesses, is crucial for optimizing treatment outcomes. NGS offers rapid, accurate and detailed microbiome analysis, enhancing patient-specific therapeutic strategies.

## Introduction

Odontogenic abscesses are potentially life-threatening infections originating from teeth or the periodontium. Possible causes include pulp necrosis, periodontitis, infected jaw cysts or inadequate chemomechanical instrumentation during endodontic treatment [1]. They account for around 4% of the number of inpatients in oral and maxillofacial surgery departments [2], therefore being one of the most common diseases in the head and neck area [3, 4].

Deterioration of odontogenic infections may lead to necrotizing fasciitis [5] or mediastinitis, resulting in high mortality rates of 10–40% [6].

Guideline-based therapy of extensive abscesses requires surgical drainage as well as additional intravenous antibiotics in an inpatient setting [2, 7]. During surgical incision, microbiological diagnostics is mandatory to analyze the pathogen spectrum and its antibiotic susceptibility. The standard method for the microbiological diagnostic is a swab and subsequent traditional cultivation of the pathogens on culture media [7]. Despite the routine use of deep wound swabs, the detection of pathogens and the associated determination of resistance to antibiotics is time-consuming, if even possible [8]. Therefore adjustment of antibiotic therapy rarely becomes a factor in patient management [9]. The reasons for this poor performance are pre-analytical errors, the considerable time it takes to obtain a result, and the basic design of conventional microbiological smear and culture diagnostics [10].

However, given the increasing rates of resistance to routinely used antibiotics [1], precise knowledge of the causative pathogens and existing antibiotic resistance are essential to improve patient outcomes and reduce complications [6, 8, 11].

Gene-based microbiological analysis methods such as next-generation sequencing (NGS) are addressing this problem. As shown in previous publications, NGS can detect a large proportion of the nearly 700 bacterial species that constitute the oral microbiome [12, 13], whereas conventional microbiological diagnostics can only detect a few microorganisms or none at all. As odontogenic infections are inherently polymicrobial [7], it is evident that this diagnostic deficit has significant influences on therapeutic outcomes.

With the growing availability and capacity of NGS testing, this study aims to investigate whether there are bacteria critical to odontogenic abscesses that can only be detected through NGS, highlighting the potential diagnostic gap between traditional and NGS microbiological testing.

## Patients and methods

### Patients

This study enrolled 51 patients who were treated for an odontogenic abscess in the Department of Oral and Maxillofacial Plastic Surgery at the University Hospital Bonn between January 2022 and April 2023. Inclusion criteria were extraoral abscess incision of abscesses originating from teeth of the lower jaw. A detailed medical history was taken from the patients before the operation. This included characteristics such as age, previous illnesses, smoking, alcohol consumption, previous treatment with antibiotics and others. In all cases, sequencable wound swabs were taken during surgical abscess incision under sterile conditions to prevent cross contamination. One conventional and one NGS smear test were always performed. After the samples had been collected, NGS swabs were sent to Zymo-Research Europe GmbH, based in Freiburg, Germany (Mülhauser Str. 9, 79110 Freiburg im Breisgau) for processing and analysis, while conventional analysis was carried out at the microbiological department of University Clinic Bonn.

All patients gave written informed consent to participate in the study before the swabs were taken. The study was approved by the responsible ethics committee of the University of Bonn (reference 447/21) and complied with the requirements of the Declaration of Helsinki.

### Methods

The sample analysis aiming for taxonomic determination of microorganisms was carried out using 16s rRNA sequencing paired with bioinformatic analysis. As part of the analyses, the microbial RNA is first extracted from the collected samples and amplified using the polymerase chain reaction. The amplifications are then sequenced using the NGS method and the identified RNA is assigned to an organism profile in a further step with the help of a curated reference database (PrecisionBIOME™ project). In this way, all bacteria and fungi present in the sample are precisely identified and quantified. In addition, the identified RNA is analyzed for the presence of the most common antibiotic-resistance genes.

The analysis detected the microorganisms at a species level. For reasons of clarity, the detected microorganisms were summarized at the genus level for analyses. If the identified genetic material could only be assigned to a family, class, or order, this is indicated. Microorganisms labeled as anaerobic are obligate anaerobic bacteria.

In addition to the gene-based analyses, data was collected from the clinical information systems, in which parameters such as the duration of treatment, previous antibiotic therapy and nicotine consumption were recorded for the subsequent data analysis. Conventional microbiological analysis and taxonomic determination was carried out as usual by cultivation of the bacteria on a nutrient medium paired with a microscopic examination. Antibiotic testing was then also carried out on nutrient media, after dilution and cultivation using antibiotic pellets.

### Statistical analysis

After completing the data collection, statistical analysis was performed using R version 4.3.1 (R Core Team, 2021, Vienna, Austria).

To analyze the difference between the absolute abundance of bacteria cells in patients with and without previous antibiotic therapy, a Wilcoxon-Mann-Whitney test was applied. For the analysis of the association between the number of bacterial genera detected in an abscess and patient characteristics such as smoking status, BMI and whether the patient received antibiotic treatment previously, regression analysis was applied. Effect estimates, 95% confidence intervals (CIs) and p values are based on ordinary least squares (OLS) univariable linear regression models. In order to investigate whether there was an association between antibiotic selection and the composition of the microbiome, the detection proportions among patients with and without previous antibiotic treatment for each bacterial genus were considered. The differences between the proportions with and without previous antibiotic treatment were than tested for equivalence between the group of aerobic and the group of anaerobic bacterial genera using the Wilcoxon-Mann-Whitney test.

## Results

### Baseline characteristics

51 Patients were enrolled in this study. The gender distribution in the cohort was balanced (male: 52.9%). The median age at abscess incision and microbiologic swab collection was 57.8 years (interquartile range (IQR): 37.8–73.1). One-third of the patients had already received oral antibiotic therapy prior to this procedure (31.4%). The most common abscess location was submandibular (39.3%) and perimandibular (37.3%). Other abscess localizations such as pterygomandibular (11.8%) or massertericomandibular (3.9%) were infrequent (Table 1). Baseline characteristics are summarized in Table 1.

**Table 1:** Baseline characteristics and frequency of different abscess localizations. Continuous variables are expressed as median with interquartile ranges in brackets, categorical variables as n and relative frequency (%). (BMI: body Mass Index; kg: kilograms; m2: square meters)

Baseline characteristics	Patients
Age at operation (years)	57.8 (37.8; 73.1)
Male gender Smoking Allergies to Antibiotics	27 (52.9%) 18 (35.3%) 5 (9.8%)
**Abscess localization**	
Perimandibular Submandibular Pterygomandibular Submental Massertericomandibular Other	19 (37.3%) 20 (39.3%) 6 (11.8%) 3 (5.9%) 2 (3.9%) 1 (2%)
Previous antibiotic treatment	16 (31.4%)
BMI (kg/m2)	28.1 (24.1; 31.2)

### Microbiological analysis

Bacteria were detected in all of the 51 swabs that were analyzed using the NGS method. Anaerobic microorganisms were found in 50 swabs, while aerobic microorganisms were detected in 30 swabs. Per swab, a median of eight genera (IQR: 6–9) were detected per swab. The median number of anaerobic genera detected was seven (IQR: 4–8), while the median number of aerobic genera detected was one (IQR: 0–1). In four cases the conventional swab was lost on transport to analysis, so the cases were excluded from comparing analysis.

The five most frequently detected bacteria were Fusobacterium (41/51), Prevotella (38/51), Parvimonas (34/51), Porphyromonas (23/51) and Streptococcus (18/51). The four most frequently detected genera were therefore anaerobes. Microorganisms that could be detected in the majority of the smears also showed the highest relative abundance (relative proportion of a bacterium in a smear). The highest abundance was observed for Prevotellaceae Family (43.9%), Prevotella (23.5%), Corynebacterium (21.8%), Porphyromonas (19.1%) and Parvimonas (16.3%). A summary of all detected bacteria can be found in Table 2.

**Table 2:** Bacterial genera detected in the NGS-Swab. Shown is the number of swabs in which the bacterial genus was detected (absolute frequency). The relative abundance indicates the percentage of bacterial RNA in the respective swab in which the bacterium was detected. Displayed as median with interquartile ranges in brackets

Bacteria	Absolute frequency (swabs)	Relative abundance (%)
Fusobacterium Prevotella Parvimonas Porphyromonas Streptococcus Eubacteriales Family XIII. Cutibacterium Peptostreptococcus Dialister Atopobium Alloprevotella Slackia Clostridiales Oribacterium Olsenella Mycoplasma Treponema Tannerella Bacteroides Campylobacter Mogibacterium Staphylococcus Lachnospiraceae Family Bulleidia Fretibacterium Solobacterium Corynebacterium Bacteroidales Order Pseudoramibacter Prevotellaceae Family Pyramidobacter Bacteroidia Class Eggerthia Pseudomonas Providencia Romboutsia Acinetobacter Brevibacterium Stenotrophomonas Micrococcus Atopobiaceae Family Desulfomicrobium Desulfobulbus Actinomycetales Saccharibacteria Johnsonella Haemophilus Gemella Rothia Carnobacteriaceae Family Neisseria Filifactor Sphaerochaeta Lawsonella Peptoniphilaceae Family Variibacter Chryseobacterium Peptococcus Family XI.	(41/51) (38/51) (34/51) (23/51) (18/51) (12/51) (12/51) (11/51) (10/51) (10/51) (10/51) (8/51) (8/51) (7/51) (6/51) (6/51) (5/51) (5/51) (5/51) (5/51) (5/51) (5/51) (4/51) (4/51) (4/51) (3/51) (3/51) (2/51) (2/51) (1/51) (1/51) (1/51) (1/51) (1/51) (1/51) (1/51) (1/51) (1/51) (1/51) (1/51) (1/51) (1/51) (1/51) (1/51) (1/51) (1/51) (1/51) (1/51) (1/51) (1/51) (1/51) (1/51) (1/51) (1/51) (1/51) (1/51) (1/51) (1/51) (1/51)	11.2 (6.7; 27.8) 23.5 (14.6; 43.0) 16.3 (8.9; 20.7) 19.1 (6.0; 36.4) 6.6 (3.7; 13.9) 6.2 (4.0; 8.0) 5.7 (3.1; 10.9) 3.2 (2.5; 7.9) 2.1 (1.4; 2.5) 6.2 (4.2; 6.9) 5.2 (2.9; 12.8) 2.7 (2.4; 3.5) 5.1 (3.9; 8.3) 3.6 (3.5; 5.1) 6.0 (4.0; 7.8) 7.2 (2.9; 15.1) 1.8 (1.7; 6.6) 5.8 (4.9; 7.0) 3.1 (2.4; 10.9) 3.2 (2.6; 6.1) 2.4 (2.0; 3.3) 6.4 (1.1; 12.7) 6.9 (4.7; 10.1) 5.6 (3.0; 8.0) 2.5 (1.9; 5.6) 2.2 (2.1; 2.8) 21.8 (16.7; 44.2) 7.0 (5.5; 8.5) 4.1 (3.4; 4.8) 43.9 3.9 2.5 6.6 6.2 4.9 4.7 3.8 3.2 2.9 2.8 6.0 6.7 5.5 2.1 5.6 3.1 8.1 4.3 3.7 3.7 0.2 2.1 4.6 0.3 0.1 0.04 6.1 3.0 5.4

**Fig. 1:**
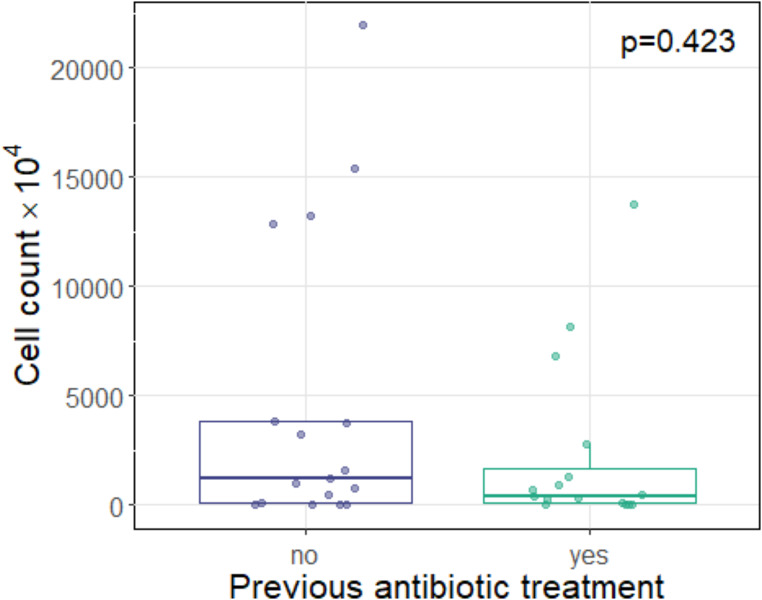
Boxplot of the absolute abundance of detected bacterial cells (cell count) depending on previous antibiotic treatment. The median cell count was lower in patients who had previously undergone antibiotic therapy (right boxplot). The difference was not statistically significant (*p* = 0.423)

The median absolute abundance of bacteria cells detected was 8.99 × 10^6^ cells (IQR: 1.06 × 10^6^-6.21 × 10^7^). Patients who had already received antibiotic treatment before swab collection exhibited a decreased median cell count (4.06 × 10^6^, IQR: 8.99 × 10^5^-1.68 × 10^7^) compared to patients without previous antibiotic treatment (1.21 × 10^7^, IQR: 9.96 × 10^5^-3.8 × 10^7^). However, the difference between these two groups was not statistically significant (*p* = 0.423; Fig. 1).

**Fig. 2:**
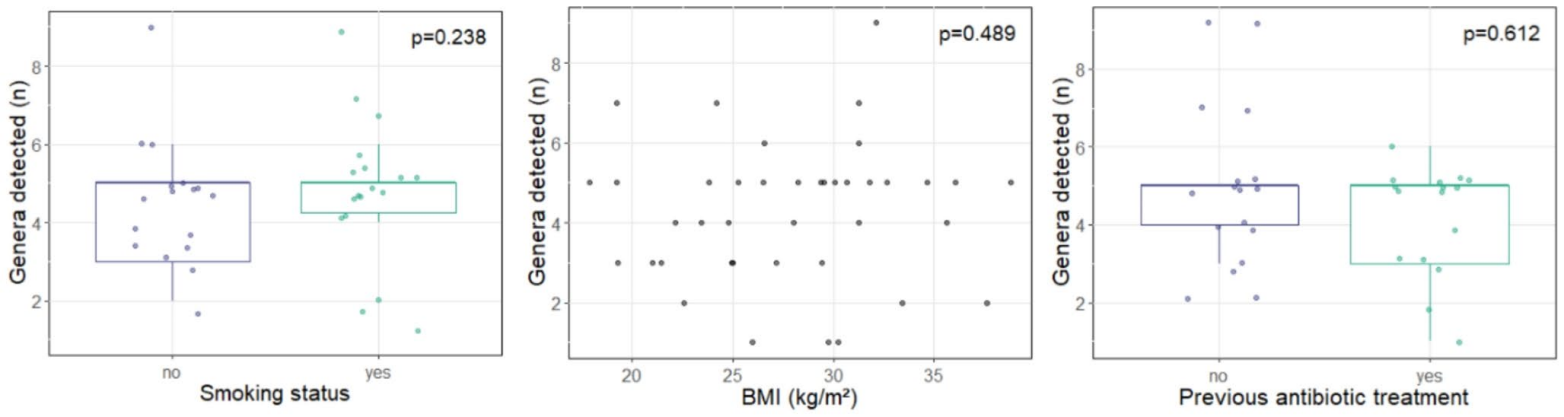
Boxplot of the number of detected bacterial genera depending on different indicators: (2**a**) Patient’s smoking status; (2**b**) BMI (kg/m2); (2**c**) Previous antibiotic treatment. The number of bacterial genera detected did not differ significantly depending on the examined indicators. (BMI: Body Mass Index; kg: kilograms; m2: square meters)

The vast majority of abscesses showed a polymicrobial infection (50/51). Only one swab showed a mono-infection with Streptococcus. The average number of bacterial genera detected in an abscess was higher in the group of patients who smoked compared to non-smokers but the difference was not significant (effect estimate: 1.26, 95% CI: [-0.87, 3.39], *p* = 0.238; Fig. 2a). In addition, no significant association between the number of detected genera and BMI was found (effect estimate: -0.06, 95% CI: [-0.23, 0.11], *p* = 0.489; Fig. 2b). Interestingly, prior oral antibiotic therapy before hospitalization also had no significant effect on the number of detected bacterial genera (effect estimate: -0.55, 95% CI: [-2.75, 1.64], *p* = 0.612; Fig. 2c).

**Fig. 3:**
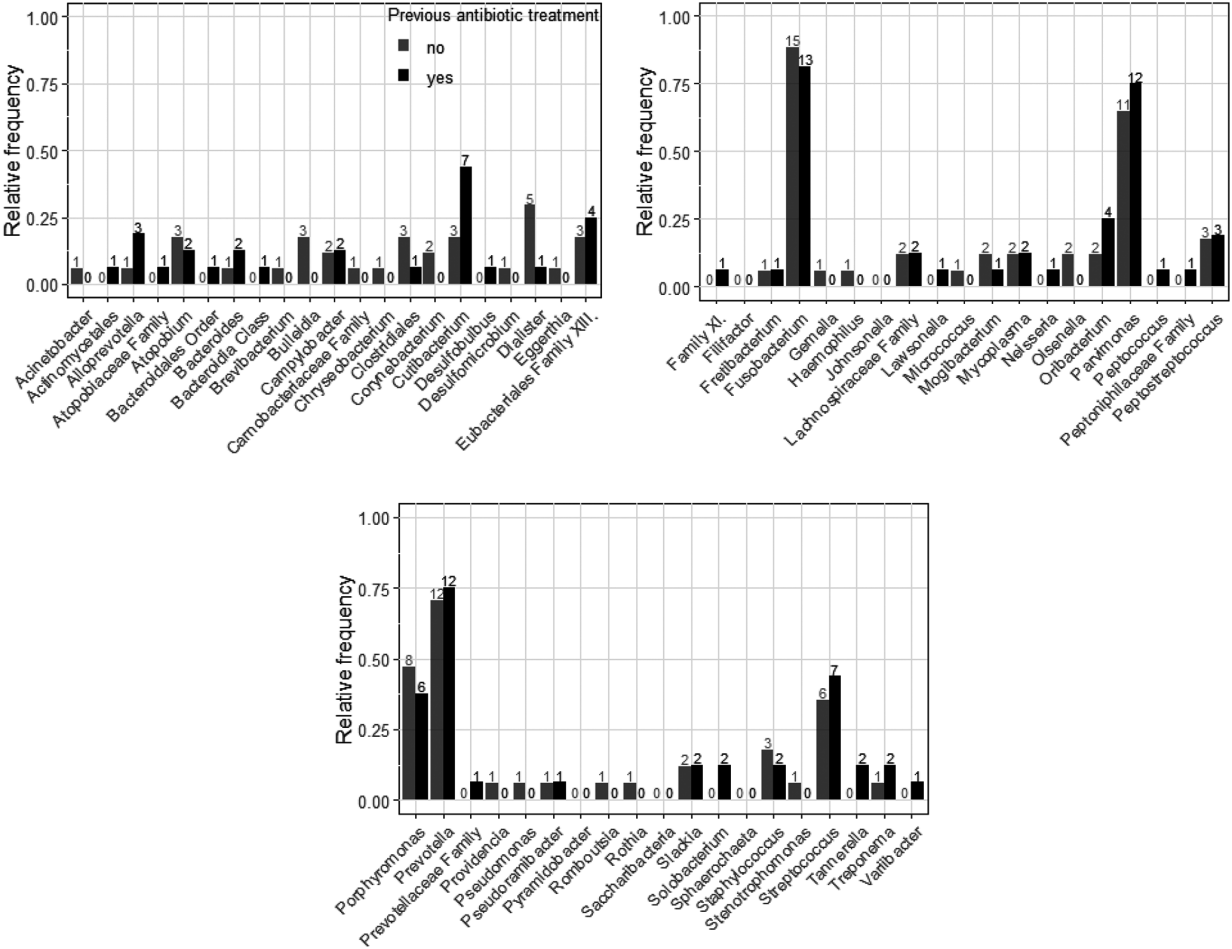
Absolute (number above the bar) and relative frequency (ordinate axis) of detected bacterial genera depending on previous antibiotic treatment. Gray: Frequency of the bacterial genus in patients who had not received prior antibiotic therapy. Black: Frequency of the bacterial genus in patients who had already received antibiotic therapy before swab collection

To investigate the influence of antibiotic selection on the microbiome, we investigated how the composition of bacterial genera differed in patients with and without previous antibiotic therapy (Fig. 3).

It was found that certain anaerobic bacterial genera were detected more frequently in patients who had already received antibiotic therapy (Table 3a), while certain aerobic bacterial genera were found more frequently in therapy-naive patients (Table 3b; *p* = 0.015).

**Table 3:** **a** Bacterial genera which were detected more frequently in patients with previous antibiotic treatment. The term delta describes the absolute increase of frequency compared to patients without previous antibiotic treatment. The term oxygen tolerance describes whether the bacteria grow under anaerobic (AA) or aerobic (A) conditions

Bacteria	Delta	Oxygen tolerance
Cutibacterium Alloprevotella Oribacterium Solobacterium Tannerella Actinomycetales Atopobiaceae Family Bacteroidales Order Bacteroides Bacteroidia Class Desulfobulbus Eubacteriales Family XIII. Family XI. Lawsonella Neisseria Parvimonas Peptococcus Peptoniphilaceae Family Prevotellaceae Family Streptococcus Treponema Variibacter	4 2 2 2 2 1 1 1 1 1 1 1 1 1 1 1 1 1 1 1 1 1	AA AA AA AA AA AA AA AA AA AA AA AA AA AA A AA AA AA AA A AA A

**Table 3:** **b** Bacterial genera detected more frequently in therapy-naive patients. The term delta describes the absolute increase of frequency compared to patients with previous antibiotic treatment. The term oxygen tolerance describes whether the bacteria grow under anaerobic (AA) or aerobic (A) conditions

Bacteria	Delta	Oxygen tolerance
Dialister Bulleidia Clostridiales Corynebacterium Fusobacterium Porphyromonas Olsenella Acinetobacter Atopobium Brevibacterium Carnobacteriaceae Family Chryseobacterium Desulfomicrobium Eggerthia Gemella Haemophilus Micrococcus Mogibacterium Providencia Pseudomonas Romboutsia Rothia Staphylococcus Stenotrophomonas	4 3 2 2 2 2 2 1 1 1 1 1 1 1 1 1 1 1 1 1 1 1 1 1	AA AA AA A AA AA AA A AA A A A AA AA A A A AA A A AA A A A

Fungi were detected in just over half of the abscesses (27/51). Malassezia (17/51), Malasseziaceae Family (5/51), Aspergillus (5/51), Malasseziales Order (4/51) and Candida (2/51) were the most frequently detected microorganisms (Table 4).

**Table 4:** Fungi detected in the NGS-Swab. Shown is the number of swabs in which the genus was detected (absolute frequency). The relative abundance indicates the percentage of mycotic RNA in the respective swab in which the microorganism was detected. Displayed as median with interquartile ranges in brackets

Fungi	Absolute frequency (swabs)	Relative abundance (%)
Malassezia Malasseziaceae Family Aspergillus Malasseziales Order Candida Fusarium Aureobasidium Chaetosphaeronema Dothideomycetes Geotrichum Annulohypoxylon Nigrospora Yarrowia Schizophyllum Hannaella Cladosporium Leotiomycetes Rigidoporus Apiosporaceae Family Phaeosphaeria Rhodotorula Coprinopsis Tremellales Order Byssochlamys Auriculariales Order Agaricomycetes Class Debaryomyces Meyerozyma Filobasidium	(17/51) (5/51) (5/51) (4/51) (2/51) (2/51) (1/51) (1/51) (1/51) (1/51) (1/51) (1/51) (1/51) (1/51) (1/51) (1/51) (1/51) (1/51) (1/51) (1/51) (1/51) (1/51) (1/51) (1/51) (1/51) (1/51) (1/51) (1/51) (1/51)	82.4 (57.1; 91.8) 5.9 (1.7; 9.1) 4.3 (2.5; 33.3) 24.6 (4.9; 54.6) 13.8 (10.0; 17.6) 25.6 (13.4; 37.8) 78.8 88.5 10.9 42.4 9.2 44.8 6.2 1.0 7.3 4.4 18.2 42.9 28.6 28.6 55.6 44.4 87.5 58.3 33.3 8.3 50.0 66.7 1.3

In addition to assigning the genetic material contained in the smears to microorganisms, an examination was also carried out for the presence of antibiotic-resistance genes. Antibiotic resistance was detected in two-thirds of the swabs examined for resistance genes (18/27). These were most frequently directed against antibiotic substance classes of lincosamides, macrolides and tetracyclines (Tables 5 and 6).

**Table 5:** Antibiotic resistance genes detected in the NGS-Swab. Shown is the number of swabs in which resistance genes were detected (absolute frequency)

Resistance gene	Absolute frequency (swabs)
tetWNW (ribosomal protection protein) ermB (ribosomal methylase) ermX (ribosomal RNA methyltransferase) mphC (macrolide phosphotransferase) msrD (ABC-F ribosomal protection protein) msrA (ABC-F ribosomal protection protein) blaZ (class A beta-lactamase) APH(3’)-Ia (aminoglycoside phosphotransferase) APH(6)-Id (aminoglycoside phosphotransferase) lnuA (lincosamide nucleotidyltransferase) APH(3’)-IIIa (aminoglycoside phosphotransferase APH(3’’)-Ib (aminoglycoside phosphotransferase) ANT(4’)-Ib (Kanamycin nucleotidyltransferase) ANT(6)-Ia (aminoglycoside nucleotidyltransferase) cmx (chloramphenicol exporter) AAC(6’)-Ie-APH(2’’)-Ia ( aminoglycoside acetyltransferase) cat (chloramphenicol acetyltransferase) sul1 (dihydropteroate synthase) mecA (penicillin-binding protein 2a) tetK (tetracycline efflux pump)	(12/27) (10/27) (7/27) (6/27) (4/27) (4/27) (4/27) (3/27) (3/27) (2/27) (2/27) (2/27) (2/27) (2/27) (2/27) (1/27) (1/27) (1/27) (1/27) (1/27)

**Table 6:** Absolute and relative frequency of antibiotic resistance in certain bacterial genera detected in the NGS-Swab (displayed only for the most common genera)

Bacterium/ Antibiotic	Amount of findings per species
**Fusobacterium**	
Clindamycin	7 (25.9%)
Macrolides	7 (25.9%)
Tetracyclines	6 (22.2%)
**Parvimonas**	
Tetracyclines	8 (29.6%)
**Streptococcus**	
Clindamycin	3 (11.1%)
Macrolides	3 (11.1%)
Tetracyclines	4 (14.8%)
Aminoglycosides	1 (3.7%)

The most frequently detected resistance genes were tetWNW (ribosomal protection protein; 12/27), ermB (ribosomal methylase; 10/27), ermX (ribosomal RNA methyltransferase; 7/27), mphC (macrolide phosphotransferase; 6/27) and msrD (ABC-F ribosomal protection protein; 4/27; Table 5). Table 6 shows which clinical antibiotic resistance could result from the identified genes for the most common genera. Conventional microbiological diagnostics revealed antibiotic resistance in 15 of 47 swabs. Resistance was most frequently found against clindamycin, glycopeptides and Amoxicillin/ Clavulanic acid (Table 7).

**Table 7:** Antibiotic resistance detected in the conventional swab. Due to the small number of detected microorganisms and antibiotic resistances, the detected resistances are not listed separately by genus. Displayed is the absolute frequency

Antibiotic	Swabs
Amoxicillin/ Clavulanic acid Clindamycin Quinolones (Moxifloxacin) Macrolides Tetracyclines Aminoglycosides Glycopeptides Penem (Meropenem) Cephalosporins (Ceftriaxone)	4 6 5 2 1 0 5 1 3

## Discussion

The management of odontogenic abscesses is a crucial aspect in the spectrum in oral and maxillofacial surgery [3]. These infections can be life-threatening and lead to complications such as intracranial abscesses [14], mediastinitis [15] or sepsis [6]. In addition, septic thrombosis of the internal jugular vein can lead to a syndromic clinical presentation known as Lemierre´s syndrome [16]. The treatment of odontogenic abscesses involves surgical incision, drainage and calculated intravenous antibiotic therapy in an inpatient setting [7, 17]. Microbiological diagnostics play a decisive role in ensuring an adequately calculated and targeted antibiotic therapy [8]. In this regard, several studies have already been carried out that analyzed the bacterial spectrum of odontogenic abscesses, using conventional microbiological culture diagnostics [9, 18–20]. This has confirmed the picture of a polymicrobial infection, which is mainly dominated by anaerobic bacteria and originates from the flora of the oral mucosa [21].

However, conventional diagnostics only detect a minor percentage of microorganisms in the abscess. Especially the detection of anaerobic bacteria and the associated determination of antibiotic resistance is not sufficient and prone to errors [22, 23]. Furthermore, results from conventional analysis seldomly influence therapy, since this technique takes several days. The clinical condition of most patients improves well under a calculated antibiotic treatment, while patients with antibiotic-resistant pathogens would benefit from a faster treatment adjustment.

Although there has been progress in the microbiological investigation of the oral biofilm in recent years, with amazing possibilities such as the evaluation of antibiotic treatment at the molecular level using electron microscopy, there is still an urgent need for a more precise breakdown of the microbiome of odontogenic infections, especially in everyday clinical practice [8, 24]. The hypothesis of this study was that the knowledge about the microbiological spectrum of odontogenic abscesses is incomplete. To give more insight into this topic a gene-based microbiological analysis using NGS was conducted.

The results of our study confirm that odontogenic abscesses are mainly polymicrobial infections [7]. At the same time, however, the results also show that the majority of the bacteria detected are anaerobes and that aerobic bacteria appear to play a subordinate role [23, 25].

About the most frequently detected anaerobic bacteria such as Fusobacterium [26], Prevotella [27] or Porphyromonas, there is a known association with periodontal disease [28], which is known to be the most common cause of the development of odontogenic abscesses [2, 25, 29]. Socransky et al. used gene-based analyses as early as 1998 to investigate which bacteria are involved in the genesis of periodontitis. They defined 5 bacterial complexes that appeared to be associated with the development of periodontitis to varying degree [28]. Bacteria with the greatest causal relationship were assigned to the red complex (e.g. Porphyromonas) or the orange complex (e.g. Fusobacterium, Prevotella). Unsurprisingly, it was precisely these pathogens that were detected with the highest frequency in the microbiome of odontogenic abscesses in our analyses. Microorganisms such as Streptococcus, which were detected at a somewhat lower frequency, belong to the yellow complex in the above-mentioned classification, which contains commensal flora of the oral mucosa that have a lower pathogenicity. Evidence of these bacterial genera in association with periodontal disease has also been found in recent studies [30].

Given the high quantity in the NGS analysis, aggressive periodontal, anaerobic pathogens seem to play a significant role in the formation of odontogenic abscesses.

The study also aimed to investigate whether the composition of the microbiome of odontogenic abscesses changes as a result of various influencing factors. It is well known that the composition of the microbiome can change due to influencing factors such as the patient’s lifestyle or an antibiotic treatment [31]. In our analyses, no significant difference between the number of bacterial genera detected in treatment-naive patients and the number detected in patients who had received previous antibiotic therapy was found.

The identified microorganisms, however, differed between the two groups. Thus, certain aerobic bacteria were detected more frequently in the group of treatment-naive patients, while certain anaerobic bacteria were found more frequently in the other cohort. This observation indicates that the administration of antibiotic substances exerts a specific selective pressure on the microbiome. It was also found that bacteria of the red complex (Tannerella, Treponema) or orange complex (Alloprevotella, Prevotellaceae), which are known to be associated with increased pathogenicity, could be detected more frequently in the group of patients who had already undergone antibiotic treatment [28]. It can therefore be argued that antibiotic therapy results in a positive selection towards periodontal pathogens and anaerobes. A different bacterial spectrum with a different resistance situation can therefore be expected in these patients, which requires an adapted, calculated antibiotic therapy during the inpatient stay. Whether this hypothesis is correct, or if we were only able to detect minor changes in the overall microbiome in this study, requires further investigation. In our current analyses, we compared only the individual detections of specific genera between the two groups. In future studies, absolute cell counts of microorganisms and their relative proportions within the total cell population identified in the smear should be compared to generate more significant results. This approach would allow for a more accurate comparison of the actual microbiome composition between the groups and facilitate the identification of relevant changes.

The role of fungi in odontogenic infections remains unclear to this day. To the best of our knowledge, this study is the first to carry out a gene-based microbiological examination of odontogenic infections and to describe the detection of mycotic RNA. In everyday clinical practice, fungi are only detected by conventional microbiological diagnostics in a very low percentage (approx. 5%) [18]. However, we were able to show that mycotic RNA was present in the smear in more than half of the patient cohort. Consequently, the low number of fungal detections appears to be mainly due to difficulties in cultural cultivation (long incubation times, etc.). Whether the microorganisms detected are clinically relevant as infectious agents or merely represent commensals or contaminants remains to be seen [32].

Taking a look at the identified antibiotic resistance genes, they mainly confer resistance to licosamides, macrolides [33] and tetracyclines [34]. The real world data from this study suggests that there could be a high number of clinical resistances to routinely used antibiotics such as clindamycin [35–40]. For example, the most frequently detected bacterial genus in the NGS analyses (Fusobacterium) showed resistance genes to clindamycin in 25.9%, while conservative culture diagnostics revealed clinical antibiotic resistance to clindamycin in 12.8% of the entire cohort. However, it should be noted that the presence of a resistance gene is not the equivalent of culturally proven clinical antibiotic resistance, as gene expression is subject to a wide variety of regulatory mechanisms. However, the presence of a resistance gene significantly increases the likelihood of resistance, compared to the absence of such genes. It therefore remains to be seen whether the gene-based identification of antibiotic resistance offers a better detection rate and should be the subject of studies with a higher level of evidence. However, when discussing a new era of microbiological diagnostics, it should also be mentioned that cultural resistance testing in everyday clinical practice often takes several days or remains unsuccessful [10, 36].

Regarding the limitations of our study, it should be mentioned that the number of patients was relatively small. This was due to the third-party funding from Zymo and is mainly due to the higher analysis costs in the past and the pilot approach of the study. In order to increase the significance and generate even greater evidence, larger randomized controlled studies will be needed for further investigation in the future. Furthermore, it should be mentioned that not all microorganisms could be identified at a genus level and there was a certain percentage of genetic material that could not be assigned to a microorganism (limit of detection). However, it is questionable, whether microorganisms in such low quantity play a significant role in a setting of odontogenic abscesses.

Nevertheless, NGS based microbiological analysis provides immediate feedback on the triggering pathogen spectrum and the existing resistance situation of odontogenic abscesses. Timely adjustment of antibiotic therapy through the use of gene-based analyses could thus influence the treatment outcome of patients in the future.

## Conclusions

This study underscores the importance of precise microbiological diagnostics in managing odontogenic abscesses, revealing the limitations of conventional culture techniques. NGS identified a predominantly polymicrobial spectrum with significant anaerobic bacteria, notably Fusobacterium, Prevotella and Porphyromonas, associated with periodontal disease. Shifts in the microbiome composition between treatment-naive and previously treated patients suggest that antibiotic therapy selects for more pathogenic bacteria, emphasizing the need for tailored antibiotic strategies.

Additionally, this study is the first to detect mycotic RNA in odontogenic abscesses, indicating a potential role for fungi in these infections. The high prevalence of antibiotic-resistant bacteria detected through NGS underscores the need for updated diagnostic practices.

The findings suggest that precise knowledge of the pathogen spectrum and antibiotic resistance can decisively influence treatment outcomes and should be incorporated into the guidelines for managing odontogenic infections, which are currently under revision.

## Data Availability

Data is contained within the article.
